# Effect of light-delignification on mechanical, hydrophobic, and thermal properties of high-strength molded fiber materials

**DOI:** 10.1038/s41598-018-19623-4

**Published:** 2018-01-17

**Authors:** Quanliang Wang, Shengling Xiao, Sheldon Q. Shi, Liping Cai

**Affiliations:** 1 College of Engineering and Technology, Northeast Forestry University, Harbin, 150040 China; 2 Department of Mechanical and Energy Engineering, University of North Texas, Denton, TX 76203 USA

## Abstract

This study developed a high-strength molded fiber material (HMFM) using pulp fibers, which could be a good substitute for plastic and solid wood materials. The surface composition, microstructure and thermal properties of HMFM were investigated by XPS, SEM and DSC, respectively. The SEM observations showed that the obvious adhesive substances and agglomeration appeared among fibers, and the inter-fiber contact area and binding tightness increased after the light-delignification. The XPS examination showed that the oxygen-rich composition on the outer surface of HMFM were reduced, and the outer surface coverage of lignin increased from 70.05% to 90.15% after the light-delignification. The DSC observation showed that the thermal stability of HMFM decreased, the temperature for the maximum rate of mass loss decreased from 370 °C to 345.6 °C, and the enthalpy value required for decomposition was reduced from 110.8 J/g to 68.0 J/g after the light-delignification. The mechanical and hydrophobic properties of HMFM were obviously improved after the light-delignification. When the content of lignin decreased from 24.9% to 11.45%, the density of HMFM increased by 6.0%, the tensile strength increased by 22.0%, the bending strength increased by 23.9%, and the water contact angle increased from 64.3°–72.7° to 80.8°–84.3°.

## Introduction

Molded fiber material is a new material developed rapidly in recent years, which can be widely used in packaging, logistics and other fields as a good substitute for plastic and solid wood material^1^. Traditional molded fiber materials are usually molded by single suction molding with a density of 0.22–0.33 g · cm^−3^, and usually used for packing small and light products^2,3^. However, high-strength molded fiber materials (HMFM) with a density of 0.8–1.1 g · cm^−3^ are molded under the hot-pressing process using plant fiber pulps as raw materials, which met the requirements of large structure and high load bearing capacity. It is possible to be applied for the large and heavy mechanical equipment packaging, collection packaging, as well as field handling^4^.

Traditional molded fiber materials take waste paper as the main raw material^5^. The main fiber type of waste paper is chemical pulp fibers, and most lignin is removed during the pulping process. The inter-fiber bonding depended mainly on the intermolecular hydrogen bonding and van der Waals forces between adjacent fiber surfaces^6^. The short and stiff fibers from waste paper were not easy to promote external fibrillation due to the repeated beating and drying^7–9^. Therefore, it is difficult to form HMFM with high density and high strength under the traditional molding process with relatively low temperature and pressure. In order to improve the physical performance of the mechanical pulp, the removal of lignin was used to form HMFM in this study^10^.

The Light-delignification refers to a certain degree of delignification treatment carried out on the high yield pulp fibers by chemical, biological or other means. In addition to the activation of the fiber surface lignin, it can also retain the lignin fragments falling off the fibers as much as possible so that they can be effectively utilized under the hot-pressing molding process to improve material performance^11–13^. Lignin is the rigid hydrophobic polymer. In the hot-pressing molding process, the retention of a certain amount of lignin may contribute to the stiffness and water resistance of HMFM^14^. Shankar *et al*.^15^ reported that the agar/lignin films exhibited higher mechanical properties compared to the neat agar film. The thermostability and waterproofness of agar/lignin composite films increased with the increased lignin content. Nakajima *et al*.^16^ observed the steep reductions occurred in the modulus of elasticity and modulus of rupture during the initial stage of delignification. Liu *et al*.^17^ stated that the addition of the enzymatic hydrolysis lignin caused an improvement in both the tensile strength and impact strength of the wood flour/HDPE composites. The water absorption and swelling of the composites decreased with the increase in enzymatic hydrolysis lignin content. Younesikordkheili *et al*.^18^ concluded that the UF resin containing lignin modified by ionic liquids had lower formaldehyde emission and water absorption content. The lignin is widely used to improve the mechanical properties, waterproofness, thermal stability and adhesive properties of fiber materials in the polysaccharide-based materials represented by wood. However, the effect of the light-delignification on the properties of HMFM has not been reported.

The light-delignification can cause the changes of the physical properties and the outer surface active groups of the fibers, which will provides a possibility for the improvement of the properties of HMFM. To improve the mechanical, hydrophobic and other properties of HMFM, HMFM before and after the light-delignification treatment were prepared via hot-pressing molding process. The chemical composition and morphology of pulp fibers before and after treatment by sodium chlorite were examined according to the standards of papermaking raw material chemical analysis and the methods of microscopic observation and mathematical statistics, respectively. The outer surface composition, microstructure and thermal properties of HMFM were investigated by the X-ray photoelectron spectroscopy(XPS), scanning electron microscopy(SEM) and differential scanning calorimetry (DSC), respectively. The effects of the light-delignification on the physical and mechanical properties (density, tensile strength and flexural strength) and hydrophobic property of HMFM were compared and analyzed. It can be used as a theoretical support for the research on the forming mechanism and optimization of the HMFM production.

## Results

### Fiber morphology and chemical composition analysis

Light-delignification can cause changes in fiber morphology and chemical composition, which in turn affected the performance of HMFM. The average and general value of the dimensions are shown in Table 1.

**Table 1:** Average and general value of the fiber dimensions.

Specimen	Statistics	Length(μm)	Diameter(μm)	Aspect ratio	Wall thickness (μm)
UTP	Mean	693.6	15.6	45.5	3.9
	Standard deviation	193.8	2.4	14.7	0.3
	General value	516.8–894.8	13.2–18.1	31.4–60.5	3.6–4.2
STP	Mean	655.8	16.9	39.9	3.7
	Standard deviation	152.4	2.6	11.4	0.3
	General value	495.5–808.3	14.3–19.3	27.7–51.9	3.5–3.9

The general value of STP fiber lengths was between 495.5 to 808.3 μm, which was smaller compared to that of UTP fibers. However, the general value of STP fiber diameters ranging from 14.3 to 19.3 μm, which was greater than that of UTP fibers. It resulted in a decrease in aspect ratio of STP fibers. The average value of fiber dimensions also showed the same changes as the general value. The fiber length decreased, the diameter increased, and the aspect ratio decreased after the light-delignification. The decrease of aspect ratio may weaken the mechanical cross-linking between fibers. However, the increase of diameter provided more contact area between fibers, which was beneficial to the improvement of inter-fiber bonding strength. The general value of STP fiber wall thickness ranged from 3.5 to 3.9 μm, which was smaller than that of UTP fibers. It may be related to the removal of lignin on the fiber surface, resulting in the decrease of fiber wall thickness and the increase of fiber softness, which was beneficial to the increase in inter-fiber compactness. Table 2 shows the yields and chemical composition.

**Table 2:** Fiber yields and main chemical compositions.

Sample	Yield (%)	Chemical composition(%)			
		Holocellulose	α-cellulose	Pentosan	Lignin
UTP	97.33	71.21	47.38	15.70	24.90
STP	77.56	80.53	53.58	16.02	11.45

The yield of STP decreased by 20.3% compared to that of UTP. In addition to the decomposition and dissolution of lignin and other compositions, it might be affected by the changes of some small fibers, which resulted in an increase in mass loss during washing. After the light-delignification, the ratio of holocellulose to lignin content varied from 2.8 to 7.0, indicating a significant change in fiber composition. The content of lignin was significantly decreased by 54.0%, and the contents of holocellulose and α-cellulose were increased correspondingly. However, the content of pentosan did not increase significantly. The Light-delignification reduced the amount of pentosan in holocellulose from 22.0% to 19.9%, showing a certain degree of degradation of hemicellulose.

### Physical and mechanical properties of HMFM

**Figure 1:**
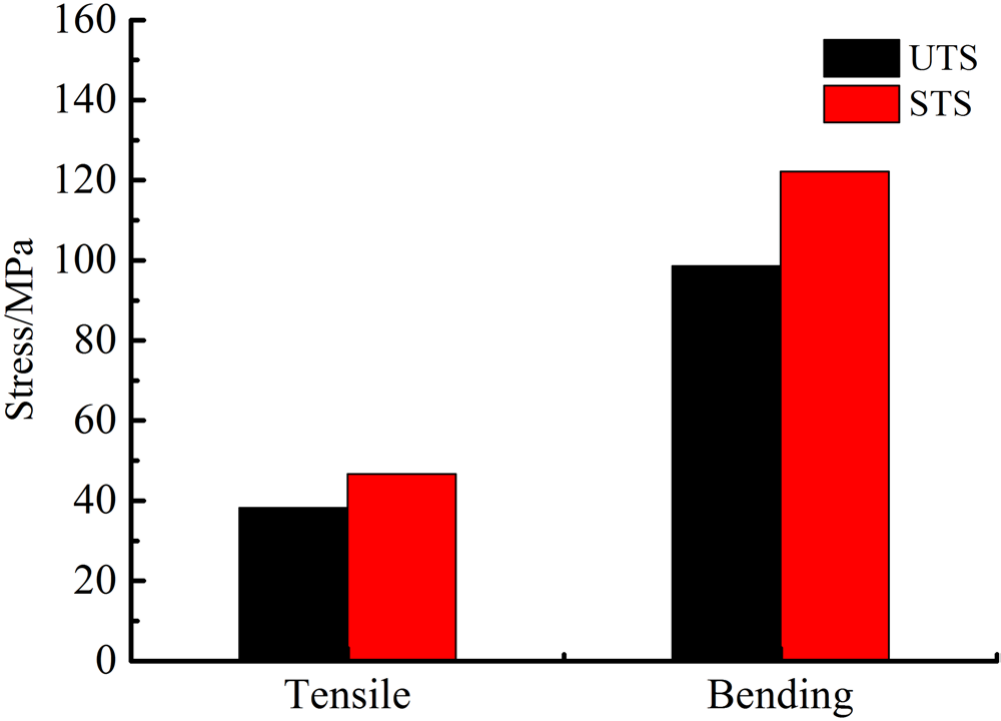
Tensile strength and bending strength of HMFM.

Mechanical strength is one of the most important indexes to characterize the performance of HMFM. The density and mechanical properties are shown in Table 3 and Fig. 1.

**Table 3:** Density and mechanical properties of HMFM.

Sample name	Density/g·cm^−3^	Tensile strength/MPa	Breaking tensile strain/%	Bending strength/MPa	Flexural strain at bending strength/%
UTS	0.986	38.24	0.79	98.59	3.29
STS	1.045	46.64	0.81	122.13	3.32

After the light-delignification, the density increased, the tensile strength and bending strength improved significantly, and the corresponding strain also increased slightly. The density of STS increased by 6.0%, the tensile strength of STS increased by 22.0% and the bending strength of STS increased by 23.9% compared to those of UTS. The light-delignification increased the softening degree of fibers. The fibers were pressed more densely, resulting in an increase in density. The formation of adhesive material between fibers, as well as the compaction of the fiber cell lumens, promoting the tensile strength and bending strength of HMFM, which was consistent with the XPS and SEM results.

### Surface chemical composition analysis of HMFM

**Figure 2:**
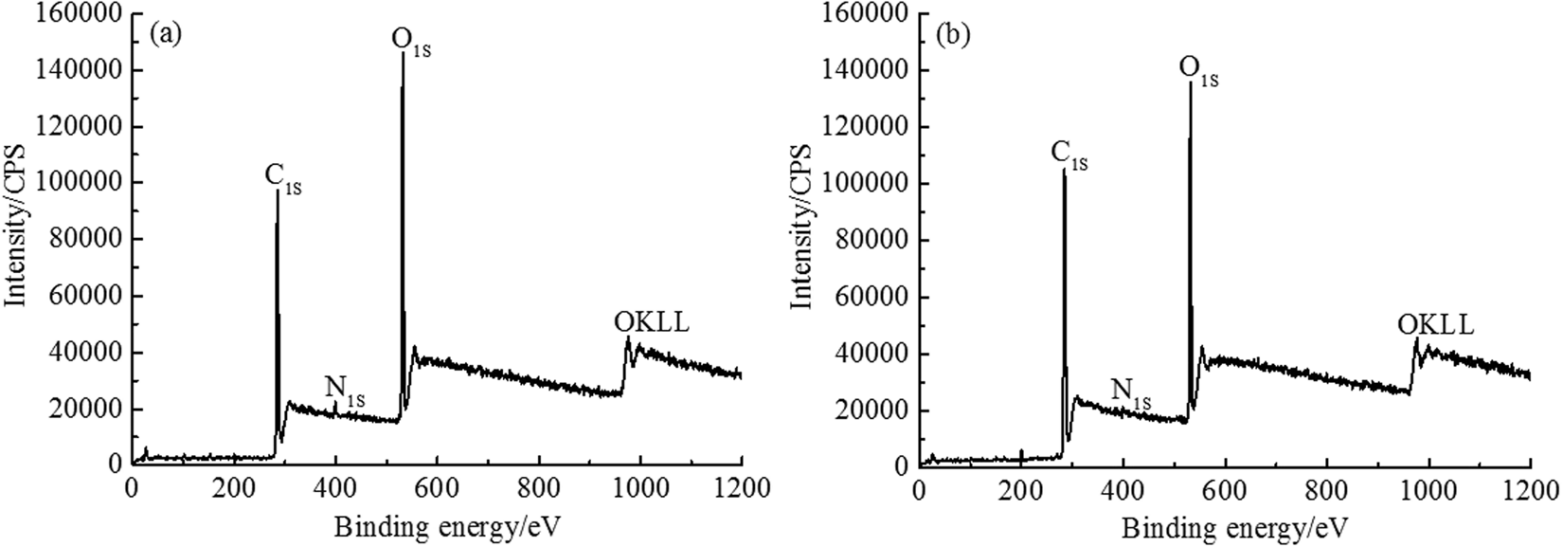
Survey spectra of samples: (**a**) UTS; (**b**) STS.

The Light-delignification caused the changes of the outer surface chemical composition of HMFM. XPS provides quantitative information of different bonded carbon atoms on the HMFM surface besides the chemical composition^19^, which are shown in Fig. 2 and Table 4. Carbon (~285 eV) and oxygen (~532 eV) were the main elements detected in the fibers in XPS survey scan, and a small amount of nitrogen (~399 eV) was also found. The outer surface nitrogen atom concentration decreased slightly after light-delignification. The outer surface lignin content of HMFM was calculated using Eq. (1), and the results are presented in Table 4. However, the outer surface lignin concentration of STS was increased. The reason may be that the light-delignification made the lignin on the outer fiber surface dissolved out in the form of debris, which was enriched on the material surface during the hot-pressing process, resulting in the increased outer surface coverage of lignin.

**Table 4:** Results of sample surface analysis by XPS.

Sample	Atomic concentration (%)			O/C	Atomic concentration (%)				C1/C2	Lignin content(%)
	C	O	N		C1	C2	C3	C4		
UTS	66.39	31.85	1.75	0.48	43.49	47.90	8.61	2.4×10^−4^	0.91	70.05
STS	71.54	27.13	1.33	0.38	54.14	36.54	8.00	1.31	1.48	90.15

The O/C ratios can be used to characterize the outer surface carbohydrate, lignin and extractives contents. Due to the removal of acetone-extracted extractives, the increase in the O/C ratio can represent a higher carbohydrate concentration on the material surface^20^. The decreased O/C ratio of the STS indicated that the oxygen-rich composition on the material surface was relatively reduced, which was consistent with the results of outer surface lignin content. The theoretical O/C ratios of cellulose and lignin are 0.83 and 0.33, respectively^21^. The O/C ratios of STS and UTS were between0.33 and 0.83, and close to 0.33, which was consistent with the chemical composition of HYP fibers.

According to the classification of carbon atoms in wooden materials, the C1s peak was deconvoluted into four subpeaks: C1 corresponds to C–C or C–H, and C1 is considered to only lignin (extractives are removed); C2 and C3 refer to the C–O and C=O or O–C–O respectively, existing in carbohydrate; C4 refers to O–C=O, which represents carboxylic acids, resins and other substances^22^.

**Figure 3:**
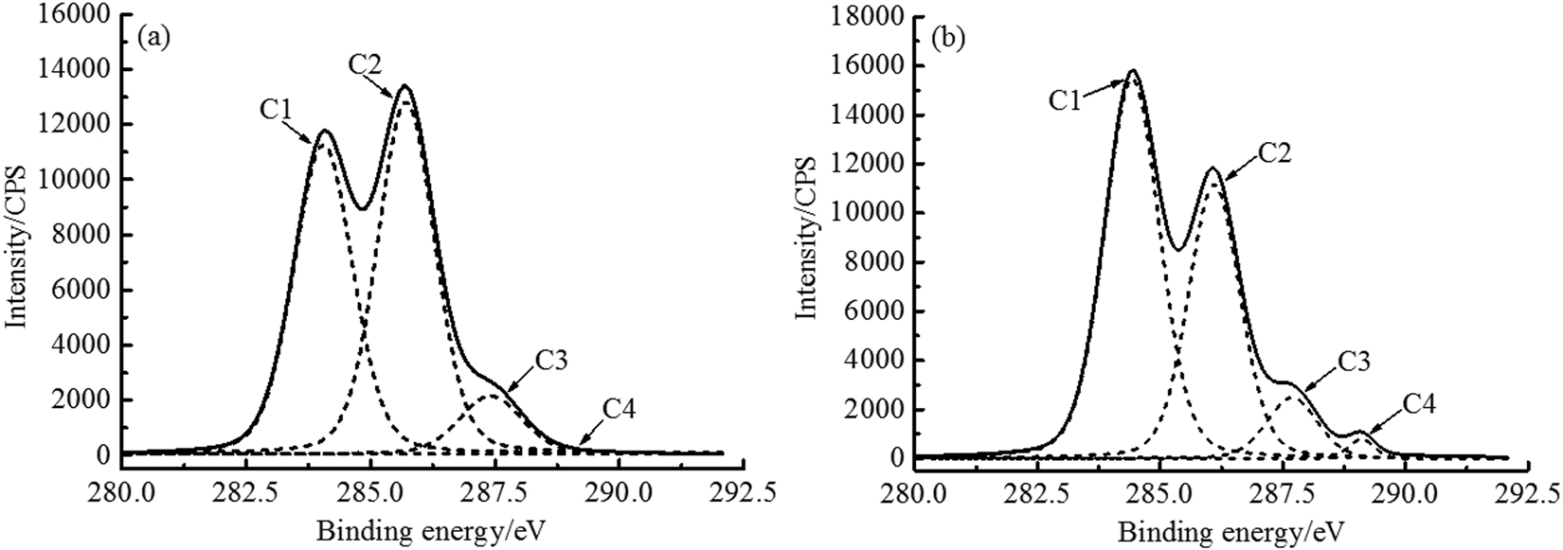
C1s spectra of samples: (**a**) UTS; (**b**) STS.

Figure 3 presents the deconvoluted C1s signals of STS and UTS. After the light-delignification, the relative amount of C1 and C4 increased obviously as C2 and C3 decreased. After the light-delignification, the degree of polymerization of lignin on the fiber surface was decreased, and more phenolic hydroxyl radicals were exposed. During the hot-pressing process, phenolic hydroxyl radicals and the degradation products of carbohydrates were polymerized, producing resin polymers useful for inter-fiber bonding, thereby the C4 relative content was increased. Additionally, the hydrophobic properties of the material surface can be expressed as the C1/C2 ratio^23^. A C1/C2 value of 0.91 was obtained for UTS, while it increased to 1.48 for STS (Table 4), indicating that the hydrophobic properties of HMFM were improved.

### Thermal properties analysis of HMFM

**Figure 4:**
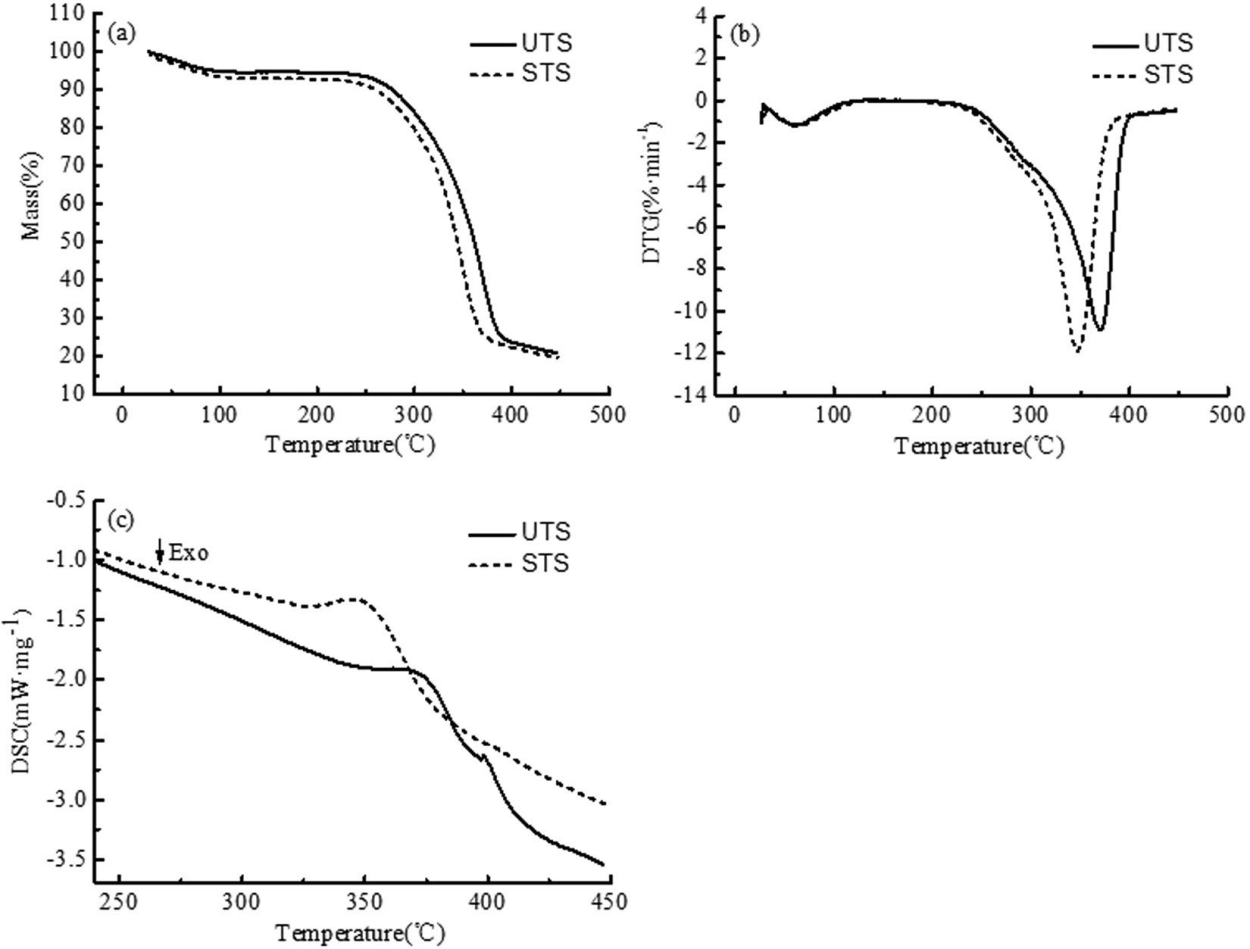
TG (**a**), DTG (**b**) and DSC (**c**) curves of UTS and STS.

The light-delignification caused changes in the fiber chemical composition, which may lead to changes in the thermal properties of the material. Figure 4 shows the TG, DTG and DSC curves of UTS and STS. Important data derived from TG curves are explained briefly in Table 5.

**Table 5:** TG results of UTS and STS. ^a^*T*_*i*_ values for initial decomposition temperature.

Samples	^a^*T*_*i*_(°C)	^b^*T*_*m*_(°C)	^c^*T*_*f*_ (°C)	RW(%), at 450 °C
UTS	242.8	370.0	394.5	20.96
STS	238.2	345.6	389.5	19.85

^b^*T*_*m*_ values for the maximum rate of mass loss. ^c^*T*_*f*_ values for the maximum decomposition temperature. RW values for residual weight.

As shown in Fig. 4(a) and (b), UTS and STS had similar weight loss behavior. From 26 to 115 °C, the weight loss was slight corresponding to the first endothermic process, which was due to the evaporation of water. But the weight loss of STS was greater, indicating that the moisture absorption was larger. From 115 to 240 °C, the samples were almost without weight loss. From 240 to 395 °C, there was a great weight loss with apparently different weight loss rates. As seen in Fig. 4(c), the characteristic peaks of the DSC curves were basically the same, indicating that similar chemical reactions occurred during the thermal decomposition process. But the intensities of the characteristic peaks were different, which was due to the difference of the content of the chemical composition.

The decomposition temperature ranges of hemicellulose, cellulose, and lignin in wood fiber materials were 180–240 °C, 230–310 °C, and 300–400 °C, respectively^24^. As seen in Table 5, The *T*_*i*_ values of STS and UTS were 238.2 °C and 242.8 °C, respectively, indicating that the hemicellulose began to undergo thermal decomposition. The *T*_*m*_ values of STS and UTS were 345.6 °C and 370.0 °C, respectively, which was due to the depolymerization of most of the cellulose and a portion of the lignin^25^. The *T*_*f*_ values of STS and UTS were 389.5 °C and 394.5 °C, respectively, indicating that the residual lignin decomposed gradually.

Compared to UTS, the *T*_*i*_ value and *T*_*f*_ value of STS decreased slightly, while the *T*_*m*_ value of STS decreased significantly. These results indicate that the STS was less thermally stable than UTS. The lignin polymerization degree of the fiber surface was reduced by the light-delignification, which weakened the barrier effect of lignin on the thermal degradation of fiber chemical composition^26^, so that the thermal stability was reduced. Compared to UTS, the *RW* of STS decreased at 450 °C, which could be attributed to the decrease of lignin polymerization degree accelerating more decomposition of lignin.

As shown in Fig. 4(c), the peak temperature values of major endothermic peaks appeared in the DSC thermogram of UTS and STS were 369.1 °C and 348.3 °C, respectively, and the corresponding enthalpy values were 110.8 J/g and 68.0 J/g, respectively. The results showed that the temperature of thermal decomposition decreased, the enthalpy required for thermal decomposition decreased and the thermal stability of material decreased after the light-delignification, which was consistent with the TG results. A weak endothermic peak in the DSC thermogram of UTS was detected at 398 °C, which was assigned to the thermal decomposition of lignin^27^. However, it did not appear in the DSC thermogram of STS, which may be related to the decrease in lignin content^28^.

### Micro morphology analysis of HMFM

**Figure 5:**
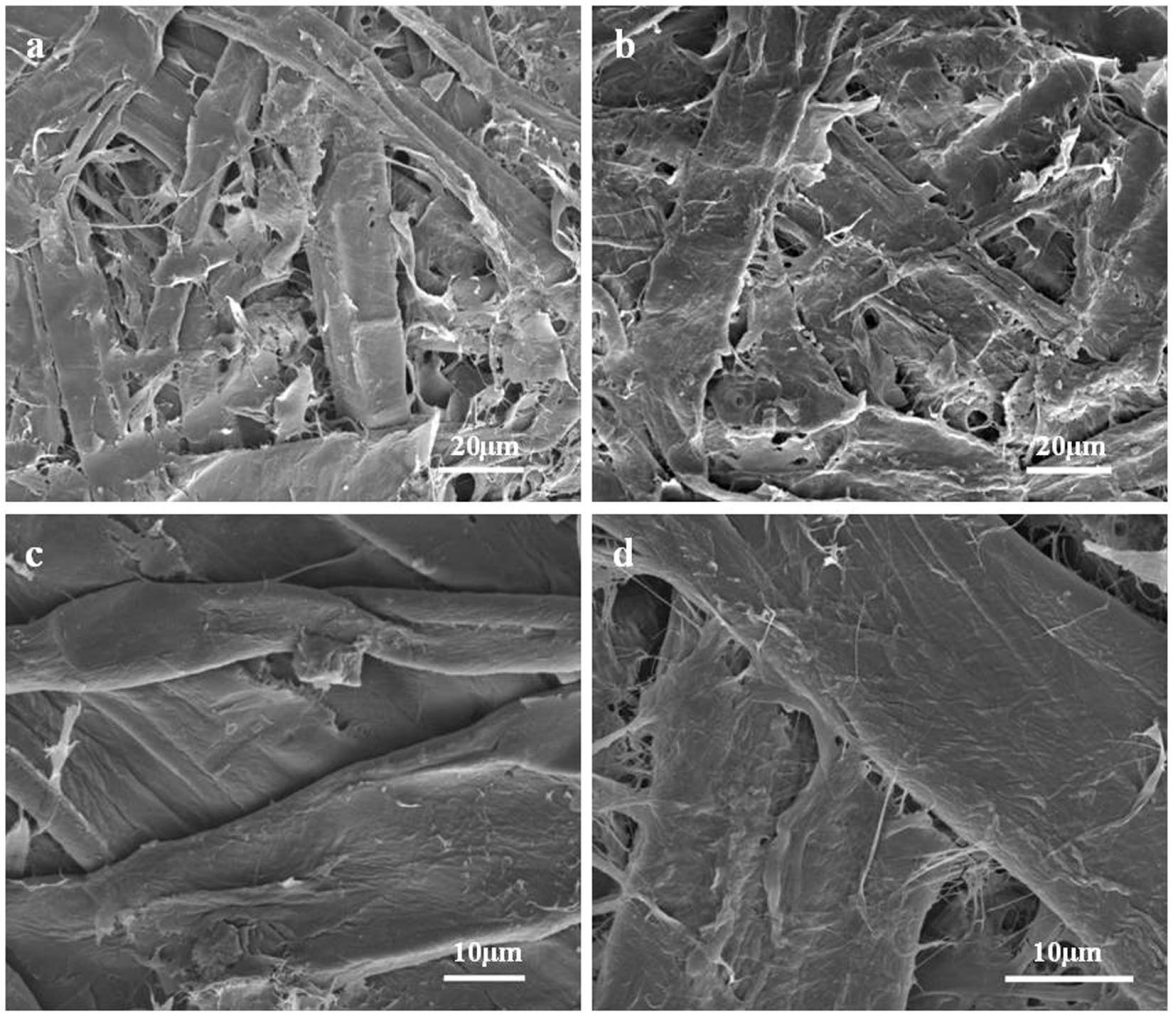
SEM micrographs of the surface of UTS (**a**,**c**) and STS (**b**,**d**).

**Figure 6:**
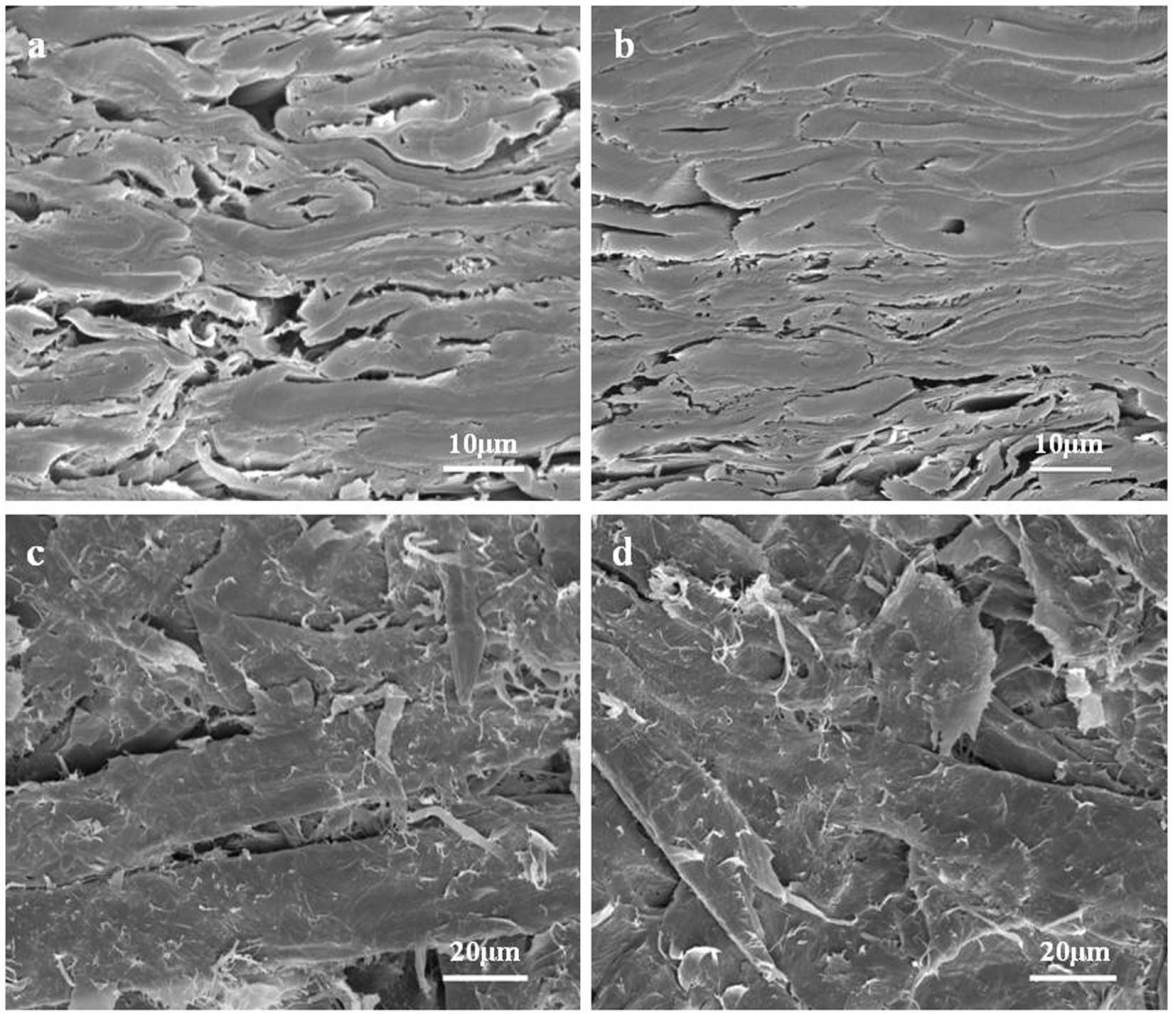
SEM micrographs of the cross section (**a** UTS, **b** STS) and inner surface (**c** UTS, **d** STS).

The light-delignification caused changes in the microscopic morphology of fibers, resulting in changes in material properties. The SEM micrographs of the material surface, cross section, and inner surface are showed in Figs 5 and 6.

As shown in Fig. 5, UTS fibers appeared stiffer, and had a lower fibrillation extent. The fibers of UTS were intertwined mechanically with smaller contact area, looser binding degree, and more surface holes. On the contrary, STS fibers showed a lower hardness. The fibers of STS bonded together by some adhesive substances with increased contact area and improved binding compactness.

As shown in Fig. 6a and b, compared to UTS, the fibers of STS appeared more compaction, more regular and neat arrangement, and increased tightness of the combination. As shown in Fig. 6c and d, compared to UTS, the fibers of STS showed more melted fragments spreading between fibers, which caused more agglomerations of fibers and contributed to the improvement of the inter-fiber bonding strength.

### Hydrophobic property analysis of HMFM

**Figure 7:**
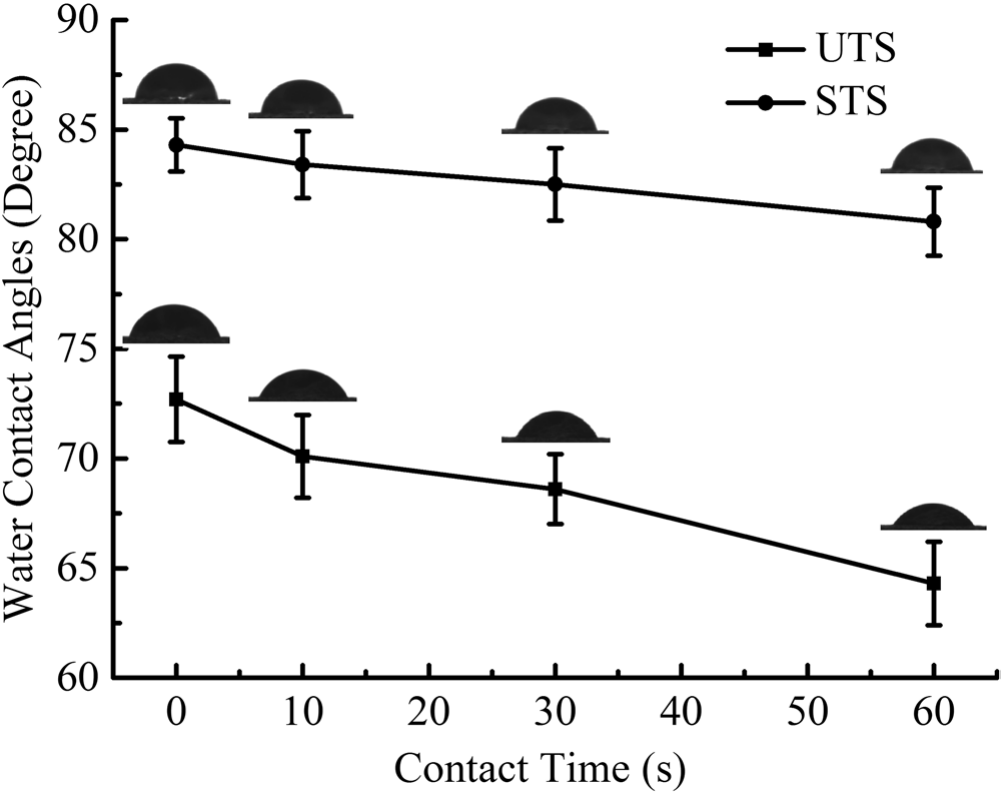
Water contact angles of the UTS and STS at different contact times.

Hydrophobic property is an important index influencing the application of HMFM. The water contact angle is an effective method to evaluate the surface hydrophobicity of material. Figure 7 shows the curve of surface water contact angle of HMFM over time.

As seen in Fig. 7, the water contact angle of UTS and STS decreased gradually over time. The water contact angle of UTS varied from 72.7° to 64.3° in 60 s, and the water contact angle of STS varied from 84.3° to 80.8° in 60 s. The significantly increased water contact angle values indicated the increase in hydrophobic property of HMFM. The stability of water contact angle over time is a very important parameter for a hydrophobic surface^29^. The water contact angle of UTS decreased 8.4° in 60 s, while the water contact angle of STS only decreased 3.5° in 60 s, which indicated that the hydrophobic stability was improved. The light-delignification significantly improved the hydrophobic property of the HMFM surface, which was consistent with the XPS results.

## Discussion

After the light-delignification, the yield of fibers decreased by 20.3%, the content of lignin decreased by 54.0%, and the ratio of holocellulose content to lignin content varied from 2.8 to 7.0, which indicated a significant change in fiber composition. After the light-delignification, the fibers showed an increase in diameter and a decrease in thickness of fiber wall, which was beneficial to the improvement of the inter-fibers bonding strength and degree of compaction, respectively.

After the light-delignification, the oxygen-rich composition on the HMFM surface was reduced, and the outer surface coverage of lignin increased from 70.05% to 90.15%. After the light-delignification, the thermal stability of HMFM was reduced, the *T*_*m*_ decreased from 370 °C to 345.6 °C, and the corresponding enthalpy values required for decomposition decreased from 110.8 J/g to 68.0 J/g. After the light-delignification, the fiber cell lumina were obviously compacted, obvious adhesive substances and more agglomerations appeared between the fibers, and the fiber contact area increased, which were beneficial to the improvement of fiber bonding strength and stiffness. After the light-delignification, the density of HMFM increased by 6%, the tensile strength increased by 22%, and the bending strength increased by 23.9%. After the light-delignification, the surface hydrophobicity of HMFM were improved, and the water contact angle increased from 64.3°–72.7° to 80.8°–84.3°. This study shows that the light-delignification can obviously improve the physical and mechanical properties and hydrophobic properties of HMFM.

## Methods

### Materials

Poplar wood chips with a size of (20–50) × (20–30) × (10–20)mm were purchased from Baihe Forestry Bureau(Jilin, China). The amount of Sodium hydroxide was 4%, the amount of anthraquinone was 0.05%, the solid-liquid ratio was 1: 4, the cooking temperature was 100 °C and the cooking time was 90 min for chemical pretreatment of poplar wood chips. Then the high yield pulp(HYP) of 40 °R was prepared by grinding in a disc refiner and defibering in a beater. All of the chemical reagents used in this study were purchased from the Sinopharm Chemical Reagent Co., Ltd(Beijing, China).

### Light-delignification treatment

50 g dry HYP was added to a beaker containing 1625 mL distilled water, and the fibers were uniformly dispersed by sufficient stirring. After adding 12.5 ml glacial acetic acid and 15 g sodium chlorite, the solution was uniformly mixed with the HYP by stirring, and the water bath temperature was maintained at 75 °C. The beakers were covered with plastic wraps to reduce the evaporation of water vapor and chlorine. After 60 min, the pulp was poured into about 10 L cold water to terminate the reaction, repeatedly washed in a 100 mesh gauze bag until neutral, balanced the moisture in the sample bag after the spin-drying, and labeled as sodium chlorite treated pulp(STP). At the same time, HYP was uniformly dispersed in pure distilled water at 75 °C and remained for 60 min, which was used as a control group and labeled as the untreated pulp (UTP).

### Morphology and chemical composition

1.0 mL fiber suspension with a mass fraction of 0.05% was placed on the glass slides. After drying, the fibers were dyed with 2–3 drops of Herzberg stain^30^. The length and diameter of fibers were observed and measured by the BX53 biological microscope (Olympus Corporation, Japan). Each group of samples were prepared with 3 pieces of glass slides containing fibers. The total number of fibers observed on each glass slide was more than two hundred. The thickness of the fibrous cell wall was measured by the cross section of the fiber in the SEM. The number of representative fibers measured for each set of samples was twenty. The general value in various sizes of fibers covered 70% of the fibers in quantity^31^.

The holocellulose, alpha-cellulose, and Klason lignin content were determined according to the ASTM standards D1104–56(1971), D1103–60 (1971), and D1106–96 (1996), respectively. The pentosan content was determined according to TAPPI T 223 CM-2010. The yields were obtained based on the ratio of the oven dry weights of the STP or UTP to the original weight of the HYP.

### Preparation of HMFM

The STP was molded in the ZT7–01molding equipment (Xingping Zhongtong Test Equipment Co., Ltd., China). The prepressing process was carried out under a pressure of 4 MPa and at a temperature of 20 °C in about 1 min to control the initial moisture content of hot-pressing at 75%. The hot-pressing process was carried out under a pressure of 8 MPa and at a temperature of 170 °C in 20 min. Both prepressing and hot-pressing processes were performed on the ZG-20T press (Dongguan Zhenggong mechanical and electrical equipment Technology Co. Ltd., China). The obtained samples of HMFM with the grammage of 800 g·m^−2^ were labeled as sodium chlorite treated samples(STS). Under the same molding process conditions descried previously, the samples of HMFM prepared by UTP were labeled as untreated samples(UTS).

### Mechanical properties of HMFM

The samples were placed at room temperature for 24 h for the density determination in accordance with ISO534: 2011. Block samples with a size of 150 × 12 mm and 100 × 25 mm were cut from HMFM for tensile testing and bending testing, respectively. The mechanical properties were examined by the CMT5504 universal strength testing machine (Shenzhen SANS Testing Machine Co., Ltd., China) in accordance with ISO527–3: 1995 and ISO 178: 2010. The average value of eight measurements in each group of samples was reported.

### Surface chemical composition of HMFM

X-ray photoelectron spectroscope data were obtained using a Thermo Fisher Scientific’s K-Alpha X-ray photoelectron spectrometer system. The Al Ka X-ray source was used. The vacuum in the analyzing chamber was 1.0 × 10^−8^ Pa during the analysis. The analyzer was operated at 50 eV pass energy for the observation of spectra. The High-resolution spectrum of the C1s region from 280 to 300 eV was collected. Elemental atomic concentrations were calculated from the XPS peak areas. Deconvolution of the overlapping peaks was performed using a mixed Lorentzian-Gaussian fitting program via XPSPEAK41 software.

Before the XPS analysis, UTS and STS were placed into the Soxhlet and extracted for 8 h with acetone. Subsequently the samples were placed on clean glass slides to evaporate the solvent, and then air-dried for 24 h. In the end, all samples were dried at 60 °C to a constant weight. After extraction with acetone, most of the extractives on the fiber surface were removed^23^.

The outer surface coverage of lignin, *ϕ*_Lignin_, was calculated at different emission angles of the electrons from the atomic oxygen to carbon (O/C) ratios^32^. The detection area of XPS was about 1 mm^2^, and the testing depth was about 5–7 nm. Assuming that the thickness of the lignin-rich regions was greater than the XPS analyzing depth, *ϕ*_Lignin_ can be estimated from the O/C atomic ratios using the following equation^33^:

1 $$\,{\varphi }_{{\rm{Lignin}}}=\frac{O/{C}_{({\rm{sample}})}-O/{C}_{({\rm{carbohydrate}})}}{O/{C}_{({\rm{lignin}})}-O/{C}_{({\rm{carbohydrate}})}}$$

where O/C_(sample)_ is the O/C ratio of the analyzed UTS or STS after extraction; O/C_(lignin)_ (0.33) is the measured O/C value in the spectrum of the lignin model compound. The extracted bleached pulp was analyzed to obtain the value for O/C_(carbohydrate)_ (0.83).

### Thermal properties of HMFM

Thermal properties of HMFM were analyzed using the differential scanning calorimeter (Diamond DSC, PerkinElmer, America). The crucibles were sealed for at least 2 h at room temperature for equilibration prior to measurement. The measurements were carried out at a temperature range of 25–450 °C and a heating rate of 10 °C/min under nitrogen atmosphere. An empty pan was used as a reference. About 8–12 mg granular sample (80 mesh) was utilized.

### SEM observations of HMFM

SEM observations were performed using a FEI Quanta-200 environment scanning electronic microscope. The specimens were coated with approximately 10 nm gold before the observation and analysis. Specimens were cut by a Feather Microtome blade to obtain a neat and smooth cross sections. Inner surfaces were obtained by tearing HMFM by hand. The observed surface was kept clean.

### Hydrophobic property determination of HMFM

The outer surface hydrophobic property was characterized by the measurement of the water contact angle on the HMFM surface using a OCA20 contact angle analyzer (Data Physics Co., Germany) at ambient temperature, and five measurements at different place of the sample were averaged and reported as the water contact angles.
